# Supplementary material for: MetaRibo-Seq measures translation in microbiomes
Source: Nat Commun. 2020 Jun 29;11:3268. doi: 10.1038/s41467-020-17081-z (PMC7324362; doi:10.1038/s41467-020-17081-z)
Supplement: Supplementary file 10 — Supplementary Data 7 [file 41467_2020_17081_MOESM10_ESM.zip › File2/Confidence_VeryHigh_Taxonomy/84450_out.krona.html]

Javascript must be enabled to view this page.

members
magnitude
magnitudeUnassigned
count
unassigned
taxon
rank

84450\_out

23

6

SRS045826\_contig\_number\_contig-100\_12324.93814SRS047044\_contig\_number\_1503SRS075716\_contig\_number\_contig-100\_20249.20250SRS144297\_contig\_number\_1160SRS893383\_contig\_number\_contig-100\_8136.117614SRS971276\_contig\_number\_contig-100\_4211.37006

superkingdom
17
2

phylum
17
1239

186801
class
17

17
order
186802

541000
17
family

genus
17
216851


SRS013476\_contig\_number\_36891SRS013965\_contig\_number\_35302SRS015431\_contig\_number\_13740SRS015782\_contig\_number\_43560SRS019068\_contig\_number\_71793SRS020233\_contig\_number\_51078SRS024132\_contig\_number\_contig-100\_1158.288286SRS049896\_contig\_number\_33047SRS049995\_contig\_number\_28124SRS063985\_contig\_number\_7853SRS077231\_contig\_number\_23283SRS097958\_contig\_number\_22472SRS143342\_contig\_number\_20873SRS144714\_contig\_number\_contig-100\_754.163720SRS148159\_contig\_number\_18620SRS148424\_contig\_number\_30824SRS893373\_contig\_number\_5127
853
species
17
